# Pathogenesis and Treatment of T-Large Granular Lymphocytic Leukemia (T-LGLL) in the Setting of Rheumatic Disease

**DOI:** 10.3389/fonc.2022.854499

**Published:** 2022-06-07

**Authors:** Nina Couette, Wael Jarjour, Jonathan E. Brammer, Alexa Simon Meara

**Affiliations:** Department of Rheumatology & Immunology, Wexner Medical Center, The Ohio State University, Columbus, OH, United States

**Keywords:** LGL, rheumatology, pathogenesis, T-LGLL, SLE (or Lupus), Behcet disease, Scleroderma (or systemic sclerosis), vasculitic, Sjogren's syndrome

## Abstract

A complex relationship exists between rheumatic diseases and cancer. This delicate balance between chronic inflammation and malignant cell transformation in hematologic neoplasms has been observed, but is not well defined. Large Granular Lymphocyte (LGL) leukemia is at the intersection of a clonal lymphoproliferative disease, chronic inflammation, and autoimmunity. The association between rheumatoid arthritis (RA) and the spectrum of Felty’s Syndrome is well-known. Other rheumatic disorders have been reported including systemic lupus erythematosus (SLE), Sjogren’s Syndrome (SS), vasculitis, Behcet’s Disease (BD) and systemic sclerosis. The association between T-LGLL and rheumatic disease pathogenesis has been hypothesized, but has not yet been fully understood. Components of a shared pathogenesis includes chronic antigen stimulation, JAK-STAT pathway activation and overlap of various cytokines. We will summarize current knowledge on the molecular understanding between T-LGLL and rheumatic disease. There are many potential areas of research to help meet this need and lead to development of targeted therapeutic options.

## Introduction

A complex relationship exists between rheumatic diseases and cancer. This delicate balance between chronic inflammation and malignant cell transformation in hematologic neoplasms has been observed, but is not well defined. Large Granular Lymphocytic (LGL) leukemia is at the intersection of clonal lymphoproliferative disease, chronic inflammation, and autoimmunity (1). LGL leukemia is a rare type of mature T cell and NK cell neoplasm that was first characterized by McKenna et al. in 1977 (2). It was given its current name following discovery of lymphocyte clonality by Loughran et al. in 1985 (3). In 1989, the French–American–British cooperative group identified LGLL as a distinct entity among T cell leukemias (4). Based on the WHO classification, this clonal proliferation can be divided into three distinct conditions: T-LGLL, chronic lymphoproliferative disorder of NK-cells (CLPD-NK or NK-LGLL), and aggressive NK-cell leukemia, of which T-LGLL is the most common accounting for 85% of cases (5). T-LGLL is frequently described in patients with rheumatologic disease (6). 15-40% of LGL leukemia patients have concomitant rheumatoid arthritis (RA) with Felty’s Syndrome representing the most well-known association (7).

Other concomitant rheumatic disorders with LGLL have been reported including systemic lupus erythematosus (SLE), Sjogren’s Syndrome (SS), vasculitis, Behcet’s Disease (BD) and systemic sclerosis (SSc), but the true frequency is difficult to assess due to the rarity of T-LGLL. There is a link in the pathogenesis between T-LGLL and rheumatic disease though the exact pathobiology underlying this has yet to be fully elucidated. Further, concomitant T-LGLL with rheumatic disease is likely underreported, as flow cytometry and testing for the T-cell receptor (TCR) are not currently standard of care for patients with rheumatic diseases. Currently, it is thought that chronic T cell activation in the setting of an antigen trigger, dysregulation of apoptosis and hyperactivation of Janus kinase (JAK) signal transducer activator of transcription (STAT) pathway as well as other molecular survival pathways (1, 8) drives the development of T-LGLL. Typical disease features of T-LGLL include splenomegaly, and cytopenias, most commonly neutropenia with increased susceptibility to infection, and anemia, often with transfusion dependence. Large granular lymphocytes bear CD3+CD8+CD57+ surface phenotypes on T cells with clonal rearrangement of TCR genes (9). These LGLs have antibody-dependent and natural killer cell-mediated cytotoxicity and make up 5-10% of total lymphocytes in healthy patients (10). Currently, treatment is based on immunosuppressive therapies, which may produce an insufficient long-term response, and make targeted therapies an ideal next step for treatment (11). Due to the rarity of T-LGLL, a significant knowledge gap exists regarding the pathogenesis and management options of T-LGLL in the setting of rheumatic disease.

The pathogenesis of LGL leukemia is thought to be due to an unknown chronic antigen trigger that leads to increased activation of the JAK-STAT pathway and emergence of a clonal population (1). Hyperactivation of the JAK-STAT pathway can be due to *STAT3* mutations that are present in 30-40% of LGL cases and mainly in patients affected by CD8+ T-LGLL subtype (12). *STAT3* mutations have been reported in patients with T-LGLL and RA (13). In a study by Rajala et al, T-LGLL patients with one *STAT3* mutation (23%) and multiple *STAT3* mutations (43%) had higher incidence of RA compared to those without mutations (6%) (14). The JAK-STAT pathway is known to play a role in the pathogenesis of other rheumatic diseases as well as provide a target for new therapies. The development of a monoclonal cytotoxic lymphocyte population is the hallmark of T-LGLL and leads to production of inflammatory cytokines resulting in disease manifestations such as cytopenias (1). Some patients with LGL leukemia can present with clinical features of rheumatic disease before the diagnosis of leukemia. It is unclear if this manifestation is related to the autoimmune disease itself or occurring as a secondary lymphoproliferative process. This review will discuss the overlap of pathogenic mechanisms and treatment between T-LGLL and rheumatic diseases other than RA.

## Chronic Antigenic Stimulation

LGL leukemia cells represent a population of cytotoxic effector memory T cells, suggesting chronic antigen stimulation (15). The role of Epstein Barr Virus (EBV), Human T-lymphotropic Virus (HTLV-1) and Hepatitis C Virus (HCV) have been suggested (1, 16–18). As in T-LGLL, various rheumatic diseases are thought to be the result of immune activation due to chronic antigen stimulation. Studies link EBV infection with autoimmune disease and some lymphoid malignancies (19). EBV has been studied extensively in RA and SLE. In SLE, the hypothesis of defective control of EBV infection in a genetically predisposed individual leads to EBV-reactive T cells, autoantibody production and resultant tissue damage (19). EBV has been found in salivary glands of patients with Sjogren’s Syndrome and EBV infected plasma cells have been shown to produce anti-Ro52 and anti-La antibodies (20). Other viral syndromes including HTLV-1, human immunodeficiency virus (HIV) and HCV share clinical features of Sjogrens (21). Currently, there is no conclusive evidence LGLs are activated by HCV, but the hypothesis of chronic self-antigen stimulation is supported by immunohistochemical studies showing LGL clusters in contact with dendritic cells in bone marrow (22). Chronic antigen stimulation from HCV has been extensively studied in the setting of cryoglobulinemia. The hepatitis C E2 envelope glycoprotein interacts with CD81 expressed on lymphocytes (23) which has been shown to result in increased T cell proliferation (24) and chronic B cell stimulation resulting in clones that produce monoclonal IgM (23), underlying the pathogenesis of cryoglobulinemia. In type II mixed cryoglobulinemia, the evolution from polyclonal to oligoclonal B cell expansion due to chronic antigen stimulation is considered to be a transition between autoimmunity and neoplasia (25). It is possible similar pathways are involved in the development of lymphoma and cryoglobulinemia in Sjogren’s Syndrome (25). LGL leukemia was associated with indolent B cell lymphoma in two patients with HCV who were successfully treated with antiviral therapy. In one case, LGL expansion correlated with viral replication and anti-viral treatment controlled LGL leukemia (26). In another example, a case of T-LGLL in a patient with concomitant hepatitis B, C and HIV was successfully treated with anti-viral therapy (27). In epidemiologic studies, HTLV-1 has increased incidence in patients with Sjogren’s Syndrome and HTLV-1 transgenic mice have shown rheumatic disease manifestations (28). The role of HTLV-1 in LGL remains unclear, but initial studies revealed HTLV seroreactivity in some LGL leukemia patients (29). In other diseases such as vasculitis, myositis and scleroderma the role of potential viral trigger is less clear and other antigenic stimulation may be result of bacterial, environmental or other triggers.

## Inherited Susceptibility/HLA Predisposition

In rheumatic diseases, the human class II major histocompatibility complex (MHC) human leukocyte antigen (HLA) plays an important role in predisposing an individual to develop an autoimmune response. Most notable is the HLA-DR region in RA, SS, SLE and vasculitis including Giant Cell Arteritis (GCA) and anti-neutrophil cytoplasmic antibody (ANCA) associated vasculitis (AAV) (30–32). In LGLL, the HLA-DR4 marker has been shown to be prevalent in patients with Felty’s/RA, but the frequency in patients with LGL leukemia that is not associated with RA is unknown (33, 34). In a small series of patients with T-LGLL, HLA-DR4 was observed in 32% of patients, in those with associated RA this was 90% (34). In another series, HLA-DR4 was highly predictive of responsiveness to cyclosporine in patients with T-LGLL supporting an immunologic mechanism underlying cytopenias (35).

## Activation of the JAK-STAT Pathway

In T-LGLL and rheumatic disease mutations of the JAK-STAT pathway play a vital role (**Image 1**). Gain of function mutations have been associated with autoimmunity as well as hematologic malignancies (36). In T-LGLL, mutation in *STAT3* gene is described most commonly leading to enhancement in anti-apoptotic pathways (37). Inhibition of the JAK pathway has been a therapeutic target for a variety of rheumatic diseases. JAK inhibitors (JAKi) have been approved for use in RA, ankylosing spondylitis (AS) and psoriatic arthritis (PsA), but studies are still ongoing for use in other rheumatic diseases such as SLE, vasculitis and SS. In T-LGLL, the JAK inhibitors ruxolitinib and tofacitinib have been applied to patients with refractory T-LGLL and related RA with some success. In a small cohort of patients receiving tofacitinib, hematologic response was observed in 67% of patients and 89% had improvement in RA symptoms (38). This has not been evaluated in cases of T-LGLL and other associated rheumatic diseases.

### Systemic Lupus Erythematosus

The role of the JAK-STAT pathway in SLE has extensively been studied with ongoing randomized controlled trials evaluating use of JAK inhibition in the treatment of SLE (39–41). (NCT03616912), (NCT03616964), (NCT03252587). It is well known the interferon (IFN) signature plays a key role in SLE pathogenesis and activation of the IFN-receptor leads to signal transduction through the JAK-STAT pathway (42). Genes including *STAT4* have been associated with high levels of IFN-alpha. This may predispose patients to SLE as overexpression of IFN-alpha genes has been found to be elevated in serum of patients with lupus (43–45). The proposed effect of STAT4 inhibition is immune suppression and inhibition of Th1 cell differentiation (42). T-LGLL is more commonly associated with *STAT3* gain of function mutation which is associated with early-onset lymphoproliferation as well as autoimmunity (46). In lupus, the role of *STAT3* has been identified in the pathogenesis of lupus nephritis. In a lupus murine model, *STAT3* knockout mice had a markedly reduced renal inflammatory infiltrate, as well as less pronounced renal IgG and C3 deposition, compared to controls (47). There has also been association of SLE development with polymorphisms in TYK2, another member of the JAK family, identified in a large Swedish and Finnish population (48). While the relationship between T-LGLL and SLE remains unclear the JAK-STAT pathway has been shown to play a role in the pathogenesis of both disease entities and may represent a potential treatment target.

### Vasculitis

The JAK-STAT pathway has also been evaluated in various vasculidites, and has been reported in patients with T-LGLL. In a series of eleven patients with vasculitis, 91% of patients had small vessel involvement presenting with purpura and histologic evidence of leukocytoclastic vasculitis. Cryoglobulinemic vasculitis was most frequently observed followed by ANCA negative microscopic polyangiitis and one case of GCA. Biopsy of the temporal artery and renal biopsy showed no LGL infiltration (49). In this series, most cases of T-LGLL were diagnosed simultaneously with vasculitis. Thus, screening for LGL in patients with new diagnosis of vasculitis should be considered.

In a study of patients with Behcets Disease (BD), total *STAT3* expression was significantly higher compared to controls, suggesting this signaling pathway is also activated (50). In a Han Chinese population with BD, a significantly increased frequency of the *STAT3* polymorphism was also observed suggesting susceptibility to BD (51). In LGLL patients, *STAT3* mutations have been associated with gene alterations on *TNFAIP3* which is a gene responsible for encoding an NF-kB signaling inhibitor called A20 (52, 53). Notably, haploinsufficiency of A20 protein can also result in a BD phenotype (54). Atas et al. hypothesized that there may be a pathogenetic association between BD and T-LGLL, due to the fact that upregulation of IL-18 and STAT3 pathways, along with a reduction in A20 protein result in reduced NF-kB inhibition (55). This overlap suggests IL-18, STAT3 and TNFAIP3 may play important roles in the pathogenesis of both BD and T-LGLL.

In large and medium vessel vasculidites, cytokine signaling dependent on JAK1 and JAK3 has been shown to be critically important in chronic inflammation (56, 57). In GCA and Takayasu Arteritis (TAK), vessel wall inflammation is induced by Th1 and Th17 cells (56). The cytokines released by these cells are known to activate the JAK-STAT pathway (36). In mouse models, temporal artery biopsy samples have shown upregulation of *STAT1* and *STAT2* genes (57, 58). A cohort study of patients with TAK revealed increased expression of various genes related to the JAK-STAT pathway (59). There are case reports of use of successful JAK inhibition in treatment of refractory TAK (60, 61).

The relationship of T-LGLL and ANCA-Associated Vasculitis (AAV) is unknown. In a cohort study of patients with AAV and nephrotic syndrome, molecular profiling of tissue samples revealed shared STAT1 activation identifying these two histopathologically different diseases have a common molecular pathway (62). Currently no clear association with *STAT3* mutations has been described in AAV. There are many unknowns for other types of vasculitis including polyarteritis nodosa (PAN) and IgA vasculitis owing to the rarity of these diseases. It is possible that advances in molecular profiling technology will increase understanding of these disease processes and identify future treatment targets.

### Sjogren’s Syndrome

In Sjogren’s Syndrome (SS), studies of JAK-STAT profiling are limited. *STAT4* polymorphisms have been identified as a genetic risk factor for SS development (63). In a study of monocytes from patients with primary SS, increased expression of JAK3 and STAT4 was detected by polymerase chain reaction (PCR) compared to controls (64). In a cohort of patients with SS, stimulation of peripheral blood monocytes by IL-6 revealed increased activation of STAT3 (65). A phenotype of LGL that has been described in association with SS represents the T_emRA_ subset, which can be seen in the setting of chronic inflammation, but is classically associated with low cell proliferation and high cell death rate compared to LGLs which have prolonged survival due to STAT pathway activation (66). Overall, these findings highlight overlap between chronic inflammation and autoimmunity as well as the difficulty associated with determining which process is the primary etiology. Further studies are needed to better assess the role of the JAK-STAT pathway in development of concomitant T-LGLL and SS. There are ongoing clinical trials evaluating the use of JAK pathway inhibition for treatment of sicca symptoms. (NCT04496960, NCT05087589, NCT04916756, NCT03100942)

### Systemic Sclerosis

Reports of T-LGLL and systemic sclerosis (SSc) are exceedingly rare. In a small cohort of patients with T-LGLL and autoimmune diseases, one patient with a diagnosis of systemic sclerosis was described (67). Cytokine analysis on T-LGLL cells was performed and showed increased levels of IL-6, IL-8, IL-10, soluble IL-12 and TNF alpha suggesting role of cytokine release related to the immune phenomena observed in LGLL (67). The JAK-STAT pathway has been shown to play a crucial role in differentiation of autoreactive cells and the extracellular matrix remodeling that occurs in SSc (68). IL-6 is thought to exert it profibrotic effect through JAK2/STAT3 signaling (69). Skin biopsies from SSc patients have also shown abnormal IL6/JAK/STAT3 and tofacitinib gene signatures (70). The role of JAK inhibition is also ongoing in clinical trials for skin and lung manifestations of SSc (NCT03274076, NCT04206644).

## Cytokines

Many cytokines involved in the pathogenesis of rheumatic disease and hematologic malignancies utilize the JAK-STAT pathway to transduce intracellular signals. Increased levels of cytokines are known to contribute to disease activity. Many different cytokines have been evaluated in the pathogenesis of T-LGLL and autoimmune disease. Leukemic LGL survival is promoted by elevated levels of IL-6 resulting in activation of STAT3 (12). Other cytokines including IL-2, IL-12, IL-15, IL-18, EGF, IP-10, G-CSF have been identified (71, 72). IL-15 has been shown to cause chromosomal instability and DNA hypermethylation acting as a key “activation switch” for survival and expansion of LGLL in both humans and mice (73). In rheumatologic disease, many cytokines use the Type 1 and 2 cytokine receptor family which has been implicated in disease pathogenesis (74, 75). The PRECISE Systemic Autoimmune Diseases (PRECISEADS) study identified a pro-inflammatory cytokine network shared by four distinct rheumatic diseases including SLE, SS, RA and SSc. Patients were found to primarily have increases in CXCL10, IL-2, IL-6, and tumor necrosis factor (TNF). The pro-inflammatory profile was also characterized by an abnormal B cell distribution, a CD8 cytotoxic T cell signature, and more severe clinical features (76). *In vitro* study suggested upregulation of this cytokine signature associated with B cell enhancement of Th1 differentiation and proliferation of activated naive T cells (76). While there is overlap between certain cytokines involved in rheumatic diseases as well as T-LGLL, whether these cytokine profiles imply a causative role is still unknown. It may be inferred that increased levels of these various cytokines support a cellular immune mechanism in rheumatic diseases and an ongoing expansion of T cells.

## Role of IL-15 in T-LGLL and Autoimmune Disease

Interleukin-15 (IL-15) is a proinflammatory cytokine expressed by a broad range of tissues and contributes to chronic inflammation and autoimmunity (77). IL-15 has been implicated in the pathogenesis of several autoimmune diseases as well as LGLL. IL-15 is a member of the IL-2 family of cytokines, which use receptor complexes containing the common gamma-chain for signaling (77). IL-15 promotes activation of T cells, NK-cells, neutrophils, macrophages, and is critical to dendritic cell function (78). Importantly related to development of autoimmune disease, IL-15 enhances activation and maintenance of IL-17 producing T Cells (75). The role of IL-15 in autoimmune disease comes extensively from studies of rheumatoid arthritis. IL-15 has been evaluated in other rheumatic diseases including SLE, SS, BD and SSc, but its exact role remains obscure (See **Table 1 and Supplement**).

Clinical trials targeting IL-15 in rheumatic disease are scarce and limited to RA. In a proof-of-concept study in rheumatoid arthritis patients, the use of human IgG1 anti-IL-15 monoclonal antibody (HuMaxIL15) showed suitable drug tolerability with no significant effects on T lymphocyte subset and NK cell numbers. By week eight, 63% of patients achieved an improvement of 20% in both the number of tender and swollen joints (79). Following, a phase II trial of the anti-IL 15 human monoclonal antibody, AMG 714, for RA did not show efficacy (NCT00433875). AMG 714 has also been evaluated in other diseases with autoimmune basis including psoriasis (NCT00443326) and celiac disease, but failed to meet its primary endpoint (80).

In T-LGLL, excess IL-15 is thought to play a part in the link between inflammation and cancer. Initial clinical trials targeting IL-15 had been unsuccessful (81, 82), but recent positive clinical data from a phase 1/2 clinical study (NCT03239392) of BNZ-1, a multi-cytokine inhibitor was presented at the 62nd American Society of Hematology (ASH) Annual Meeting suggests that IL-15 inhibition can induce clinical responses in patients with T-LGLL, particularly those with transfusion dependence (83).

## Linking Autoimmunity and Cancer: IL-15 Regulatory Pathways

A common feature of CD8+ T cells and NK cells is their dependence on IL-15 for homeostasis (84, 85). Zhou et al. describe the deubiquitinase, Otub1 which was shown to be a key regulator of IL-15R signaling. Otub1 deficiency was associated with anti-cancer immunity and loss of self-tolerance (86). This highlights the role of Otub1 as a potential novel checkpoint target for cancer therapy. Other clinical trials using IL-15 in treatment of cancer have shown increased activation of NK and CD8+ T cells, but when administered as monotherapy have been ineffective (87). This is thought to be due to the action of immunologic checkpoints and there are ongoing trials evaluating the use of IL-15 in combination with checkpoint inhibitors for patients with metastatic solid cancers (NCT03388632). Combination therapy of IL-15 with rituximab in a mouse model of lymphoma and alemtuzumab in a model of adult T cell leukemia revealed that IL-15 enhanced efficacy of both rituximab and alemtuzumab (88). This led to development of the phase 1 trial of IL-15 combined with alemtuzumab for patients with adult T cell leukemia (NCT02689453) as well as ongoing trials in chronic lymphocytic leukemia (NCT03759184, NCT03905135).

## Role of Other Cytokines in T-LGLL and Rheumatic Disease

### Systemic Lupus Erythematosus

SLE has been considered a dominant Th2 cytokine disease though, increased levels of both Th1 and Th2 cytokines can be seen (89). An association between IL-18, SLE and T-LGLL has been proposed. IL-18 is a cofactor for Th1 cell development and cytotoxic T cell induction (90). Ogata et al. describe a case of SLE and T-LGLL with levels of IL-18 correlating with lupus symptoms as well as the number of T-LGLs in serum suggesting IL-18 may activate T-LGLL (91). In a study of 40 patients with SLE, plasma IL-18 and IL-12 concentrations were significantly higher in SLE patients than in controls (92). In mouse models, CD8+ cytotoxic T cells have been found to be elevated in IL-18 transgenic mice and aberrant expression of IL-18 resulted in the increased production of both Th1 and Th2 cytokines (90). The MRL/lpr mouse, used as a clinical model in SLE, has been found to have higher serum levels of IL-18 compared to wild-type mice (93). In the same study, injections of IL-18 lead to presentations of malar rash and glomerulonephritis. This highlights the important role IL-18 plays in SLE and possibly the development of T-LGLL, but also as a potential therapeutic target.

### Sjogren’s Syndrome

Levels of different cytokines in association with T-LGLL and SS have been evaluated in a series of 12 patients which revealed significantly increased levels of soluble interleukin-2 receptor, TNF-alpha, IL-6 and IL-8 compared with healthy controls (94). This increase was common to LGL leukemia patients with or without Sjogren’s syndrome.

### Vasculitis

Cytokine profiles in vasculitis vary based on the specific underlying diagnosis and the connection with T-LGLL is still not clearly characterized. In large vessel vasculitis such as GCA, key cytokines identified include IFN-gamma, IL-6, IL-12, IL-17, IL-18 and IL-21 (56, 95) which promote Th1 and Th17 cell differentiation (96). In patients with granulomatosis with polyangiitis (GPA), monocytes have been shown to release high levels of IL-12 leading to induction of Th1 cytokines including TNF-alpha and IFN-gamma (97). In Behcet’s Disease, most studies have shown evidence of a Th1 predominant response, but Th2 and Th17 involvement have also been demonstrated (55). Levels of IL-2, IL-12, IL-18 and IFN-γ (Th1 proinflammatory cytokines) have been shown to be increased in BD (98) and elevated levels of IL-18 have also been linked with disease activity (99).

### Systemic Sclerosis

Increased levels of IL-1, IL-2, IL-2R, IL-4, IL-8, IL-17, TNF-alpha, interferon, and antibodies to IL-6 and IL-8 have been found in sera of patients with SSc (100, 101). The role of IL-6 has been highlighted as increased levels have been linked to more severe skin and lung disease (102). The IL-6 inhibitor, tocilizumab is approved for use in SSc related interstitial lung disease. While a variety of cytokines are involved in autoimmunity and malignancy the question of whether anti-cytokine therapies may play a preventative role in T-LGLL is unknown. Chronic stimulation by proinflammatory cytokines including IL-6 is responsible for sustained LGL proliferation as well as an important *STAT3* activating factor (103). Studies have revealed increased levels of IL-6 in plasma of patients with LGLL compared to healthy controls (67, 104). IL-6 inhibitors are also used as treatment for other rheumatic conditions including GCA, RA and Castleman disease, but its role as use for prevention or treatment of T-LGLL is lacking clinical data. Based on the role of IL-6 in pathogenesis of LGLL, there has been consideration to use of tocilizumab as salvage therapy in T-LGLL (105). In addition to anti-cytokine therapies, similar questions arise for the role of JAK-STAT inhibitors, as this pathway plays a central role in LGLL pathogenesis. This class of drugs is more commonly being used to treat inflammatory arthritis, but due to lack of clinical data the role as preventative therapy for T-LGLL is lacking and it is unknown if patients with inflammatory arthritis treated with these drugs are less likely to develop LGLL.

## Role of Sphingolipids in T-LGLL and Rheumatic Disease

Sphingolipids have been shown to play a part in long term survival of cytotoxic lymphocytes (106). Dysregulation of the sphingolipid pathway in rheumatic diseases has rarely been described. In SLE, a cohort study revealed clinical and renal disease activity were associated with elevated levels of circulating sphingolipids (107). In another study of patients with biopsy proven lupus nephritis, serum levels of sphingolipids were higher compared to controls (108). As dysregulation of pro-apoptotic (ceramide, sphingosine) and pro-survival sphingolipids (sphingosine-1-phosphate) has been shown to play a role in T-LGLL (106, 109) it would be of interest to evaluate the value of sphingolipids in patients with rheumatic disease.

## Treatment:

### JAK Inhibitors in the Management of T-LGLL and Rheumatic Disease

The discovery of JAKs as targeted therapy led to improvements in treating many rheumatic diseases including RA, polyarticular juvenile idiopathic arthritis (JIA) and psoriatic arthritis. There are currently three JAK inhibitors (JAKi) approved for use in patients with rheumatic disease in the United States. Tofacitinib, baracitinib and updacitinib are approved for use in active RA in patients who have had inadequate response to methotrexate, traditional disease modifying anti-rheumatic drugs (DMARDs) and tumor necrosis factor inhibitors (TNFi). Tofacitinib is also approved for use in polyarticular JIA, psoriatic arthritis and ankylosing spondylitis. The pan-JAKi, Peficitinib is approved for RA in Japan, South Korea, and Taiwan (110). Filgotinib, a Jak 1 inhibitor is approved for RA in Japan and Europe (111).

It can be speculated that due to improvements in earlier RA diagnosis and initiation of treatment this may lead to an overall decrease in clonal expansion and development of T-LGLL. Many therapeutic options are available for RA, but their specific role in driving clonal expansion is unknown. In a study of 529 patients with RA, 19 (3.6%) patients exhibited T-LGL expansion. There was a significant association with the T-LGL clone and duration of TNF inhibitor use suggesting long term exposure may be associated with increased clonal T-LGL cells in RA patients (112). Similar results were demonstrated in a cross-sectional analysis of patients with psoriatic arthritis and ankylosing spondylitis (113). A variety of *in vitro* and murine studies have shown mechanisms of potential benefit for use of JAK inhibition in rheumatic diseases including SS, SLE, large vessel vasculitis, dermatomyositis and SSc though overall data is limited. Most clinical evidence comes from case reports however there are ongoing randomized trials with a variety of JAKi for other rheumatic disease indications.

In Sjogren’s Syndrome, a phase II trial of filgotinib failed to meet its primary endpoint (NCT03100942) and there are ongoing trials evaluating the use of tofacitinib and baracitinib. Notably in SLE, a phase 2 trial of baricitinib was successful in patients with active skin and joint disease and phase 3 trials are ongoing (41). Evidence for use of JAKi in vasculitis is scarce. Most data from *in vitro*, murine models and clinical experience suggest a pathogenic basis that JAKi may be beneficial, but clinical trials are needed. Data has come primarily from studies involving large vessel vasculitides such as GCA and TAK (36). There are ongoing clinical trials evaluating the efficacy of JAK inhibitors in both of these diseases (NCT04299971, NCT03026504, NCT03725202, NCT04161898). In other vasculitides such as Behcet’s and Polyarteritis Nodosa, JAKi has been reported in cases of refractory disease with some success (114). In a study of 13 patients with refractory BD, patients who were treated with tofacitinib showed improvement in vascular and joint symptoms (115). A pilot study of 10 patients with AAV treated with tofacitinib were found to have improvements in clinical symptoms and reduction in steroid requirements (116), but larger randomized trials are needed to confirm these findings. There are also ongoing trials of use of JAKi in SSc and dermatomyositis (NCT03274076, NCT03002649, NCT04966884, NCT04613219).

The role of JAK inhibitors as targeted therapy in T-LGLL associated with rheumatic disease is not known. In a study of nine patients with rheumatoid arthritis and refractory T-LGLL, tofacitinib led to hematologic response in six patients and improvement in synovitis in eight patients (38). This may suggest a role for earlier use of JAKi in patients with concomitant RA and T-LGLL, but larger studies are needed. JAKi use in other rheumatic conditions associated with T-LGLL have not been reported.

The use of JAKi in T-LGLL is currently being evaluated, though early promising data from a Phase I basket study suggests there may be some efficacy. Targeted therapy with Ruxolitinib, a JAK 1 and 2 inhibitor, was evaluated in five cases of refractory T-LGLL with partial response observed in two patients, and improvement in cytopenias in 4 patients (117). There is an ongoing trial of Ruxolitinib in relapsed or refractory T or NK cell lymphoma (NCT02974647) and this study is being evaluated in a multi-center phase II trial. Ruxolitinib safety, tolerability and efficacy was also evaluated in a four-week trial in patients with RA (NCT00550043), but there are no published results. Another targeted therapy, BNZ-1, is a multi-cytokine inhibitor that targets the gamma chain receptor subunits of IL-2, IL-9, and IL-15 leading to reduction of cytokine-mediated cell survival (118). First clinical data with BNZ-1 in LGL was completed in a phase I/II trial with 20% ORR (3PR, 1 CR), particularly in patients with transfusion-dependent anemia (83). In regard to other autoimmune disease, there is a phase II trial ongoing for alopecia, but no other active trials in rheumatic disease at this time (NCT03532958).

While standard therapies used in symptomatic T-LGLL include steroids, methotrexate, cyclosporine and cyclophosphamide, these are effective in only 30-40% of cases (11, 119). No clear treatment guidelines have been established due to a lack of clinical trial data. In patients with T-LGLL and associated rheumatic disease co-management with a rheumatologist is key. Treating the underlying rheumatic process may be the best initial step to alleviate T-LGLL. While methotrexate is often a first line therapy in the setting of inflammatory arthritis and other rheumatic diseases, initial treatments used in T-LGLL including cyclophosphamide are often reserved for severe organ or life-threatening manifestations of rheumatic disease. There is a clear need to develop better therapies for the treatment of T-LGLL and T-LGLL in the setting of rheumatic disease.

## Summary

Chronic inflammation and immune activation are central to the bidirectional relationship between cancer and rheumatic disease. Components of a shared pathogenesis between T-LGLL and rheumatic disease includes chronic antigen stimulation, JAK-STAT pathway activation and overlap of various cytokines. Due to the rarity of T-LGLL in the setting of rheumatic disease this complex relationship remains difficult to define. It is important to evaluate the presence of T-LGLL in patients with rheumatic disorders, as T-LGLL is likely under-reported in this population. While T-LGLL and rheumatic conditions may share clinical and lab features, a complete history and examination by a rheumatologist is key for appropriate serologic evaluation and diagnosis of rheumatic disease. In the setting of cytopenia, early evaluation with peripheral blood flow cytometry and TCR testing would likely improve recognition and early detection of T-LGLL.

## Author Contributions

NC wrote the first draft of the manuscript. AS, JB, WJ contributed to manuscript revision, read and approved the submitted version.

## Conflict of Interest

The authors declare that the research was conducted in the absence of any commercial or financial relationships that could be construed as a potential conflict of interest.

## Publisher’s Note

All claims expressed in this article are solely those of the authors and do not necessarily represent those of their affiliated organizations, or those of the publisher, the editors and the reviewers. Any product that may be evaluated in this article, or claim that may be made by its manufacturer, is not guaranteed or endorsed by the publisher.
