# Supplementary material for: Pathogenesis and Treatment of T-Large Granular Lymphocytic Leukemia (T-LGLL) in the Setting of Rheumatic Disease
Source: Front Oncol. 2022 Jun 7;12:854499. doi: 10.3389/fonc.2022.854499 (PMC9209697; doi:10.3389/fonc.2022.854499)
Supplement: Supplementary file 1 [file DataSheet_1.docx]

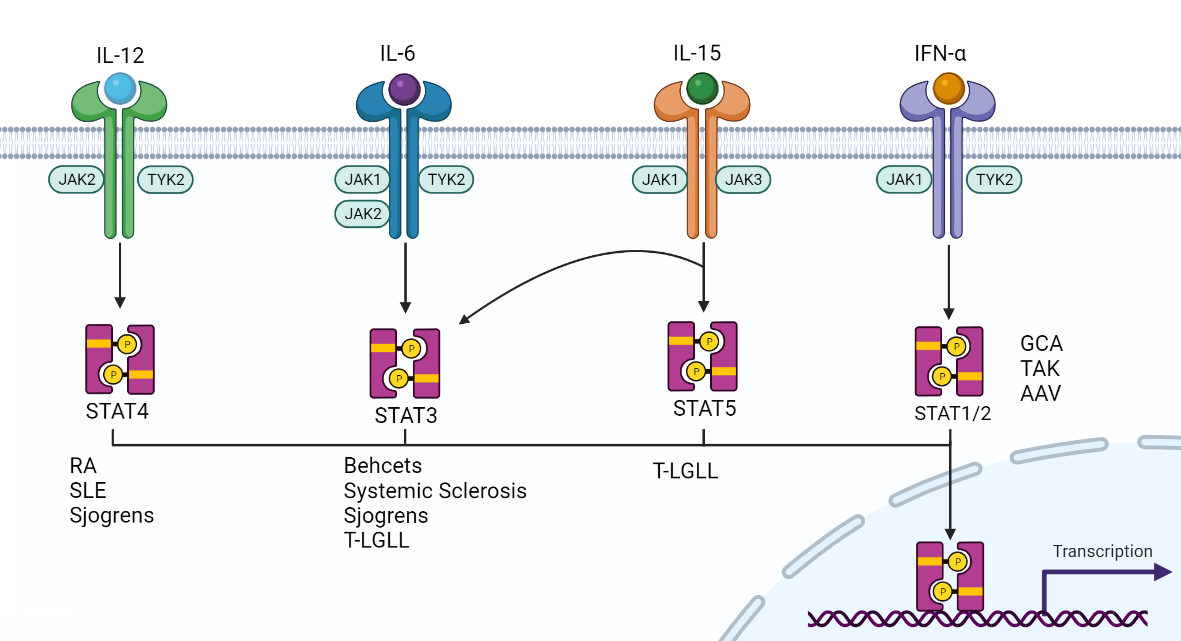


**Image 1:** Pathways that lead to STAT activation in autoimmune rheumatic disease and T-LGLL. Created on biorender.com

Legend: Rheumatoid Arthritis (RA), Systemic Lupus Erythematosus (SLE), T-Large Granular Lymphocytic Leukemia (T-LGLL), Giant Cell Arteritis (GCA), Takaysu Arteritis (TAK), ANCA-Associated Vasculitis (AAV)
