# Supplementary material for: Pathogenesis and Treatment of T-Large Granular Lymphocytic Leukemia (T-LGLL) in the Setting of Rheumatic Disease
Source: Front Oncol. 2022 Jun 7;12:854499. doi: 10.3389/fonc.2022.854499 (PMC9209697; doi:10.3389/fonc.2022.854499)
Supplement: Supplementary file 2 [file Table_1.docx]

| **Study** | **Disease** | **IL-15 Relevance** | **Results** | **P=value** |
| --- | --- | --- | --- | --- |
| Wuttge (2017) | SSc | Increased serum IL-15 levels in SSc patients vs. healthy controls (HC)  IL-15 levels did not differ between limited and diffuse SSc. | HC: (0 (0 to 0.46) pg/ml)  SSc: (0.63 (0.47 to 0.88) pg/ml) | (p < 0.01) |
|  |  | High IL-15 (>0.88 pg/ml)) correlated negatively with the VC and DLCO | VC: 66–89%  DLCO: 62–81% | VC (p < 0.05)  DLCO (p < 0.05) |
| Gholijani (2017) | BD | Serum IL-15 levels were elevated in BD vs. HC | HC: 0.87 pg/ml +/- 0.16  BD: 1.11 pg/ml +/- 0.09 | (p < 0.001) |
| Choe (2013) | BD | Serum IL-15 levels in BD patients are higher than those of HC, SLE, and RA patients | 10.4±0.9 pg/ml in HC  10.9±1.0 pg/ml in SLE  13.7±1.0 pg/ml in RA,  16.3±1.5 pg/ml in BD patients | HC: (p < 0.001)  RA: (p < 0.001)  SLE: (p < 0.001) |
| Hamzaoui (2006) | BD | Active BD patients have higher serum IL-15 levels compared with BD in remission and HC | HC: (4.65 pg/ml; 3.9-6.2).  Active BD: (median 10.4 pg/ml; range 5.3-17.4)  BD in Remission: (6.05 pg/ml; 4-10.4) | (p <0.02) |
| Madej (2015) | SpA | Elevated IL-15 serum levels in axial SpA compared to HC. | SpA group: 1.96 ±0.66 pg/mL  HC: 0.02 ±0.06 pg/mL | (p=0.0000) |
| Chan (2008) | SpA  RA  OA | Expression of IL-15 on fibroblast-like synoviocytes was higher in SpA and RA patients compared to OA. | SpA: (mean SD 6.8 +/-1.8% [n=6])  RA: (8.9 +/-1.5% [n=4])  OA: (2.3+/-0.9% [n=8]) | (p=0.005 for both comparisons) |
|  |  | Surface IL-15 expression increased upon coculture with NK cells | SpA: 16.9 +/- 2.2%  RA: 20.9 +/- 3.3%  OA: 4.1 +/-1.4% | (p=0.001 for SpA or RA vs OA) |
| Kane (2004) | PsA | IL-15 gene expression in synovium of PsA patients | Before Methotrexate: 46.9 +/- 59.8  After Methotrexate: 15.8 +/- 16.6 | (p=0.33) |
| Sisto (2017) | SS | The level of IL-15 mRNA was expressed in lower amounts in SS salivary gland epithelial cells (SGEC) treated using blocking antibody to TLR2 than in untreated pSS SGEC | The expression of IL-15 protein was decreased from 93.48±2.4 % of the pSS SGEC value to 63.8±1.2 % of SS SGEC treated with anti-TLR2 antibody. | (p=0.01) |
| Reksten (2009) | SS | IL-15 levels elevated in SS with Focus Score (FS) >1:  (FS+) and FS < 1 (FS-) and in patients with Germinal Center (GC) formations (GC+) and without. | FS-: 273 +/- 139 pg/mL  FS+: 323 +/- 92 pg/mL  GC-: 172 +/- 46 pg/mL  GC+: 715 +/-3 pg/mL | GC: (p < 0.01) |
| de Menthon (2011) | GPA | IL-15 levels were higher in serum of GPA patients than HC | See study figure 3B. | (p<0.0001) |
|  |  | IL-15–induced expansions of NKG2D+CD4+T cells were larger in GPA patients than in HC | See study Fig. 3A | (p=0.0006) |
| Lugo (2019) | SLE- Renal | Lupus nephritis patients had increased IL-15 compared to the SLE non-nephritis group | See study Fig. 2 | (p=0.0084) |
| Lin SJ (2017) | SLE | SLE patients with active disease show higher IL-15  serum levels compared to inactive patients | Inactive: 8.6±1.0 pg/ml  Active SLE: 19.4±4.1 pg/ml | (p = 0.019) |
|  |  | IL-15 increased NK cytotoxicity of active SLE patients and inactive SLE. | Inactive SLE: 20.1±4.0% vs. 5.1±1.2%,  Active SLE: 13.8±2.9% vs. 3.8±0.8%, | Inactive: (p = 0.003)  Active: (p<0.001) |
|  |  | IL-15 enhanced CD69 expression on NK cells of patients with inactive disease and patients with active SLE. | Inactive disease: (26.5±4.7% vs. 5.0±1.1%  Active SLE disease: (28.7±4.4% vs.  7.8±1.6% | Inactive: p<0.001)  Active: p<0.001) |
| Lin SJ (2016) | SLE | IL-15 enhanced CD11b expression of NK cells from  SLE patients | (50.1 ± 2.9% vs 42.9 ± 3.1%) | (p = 0.012) |
|  |  | IL-15 enhances expression of CD54 on NK cells from SLE patients | (27.4 ± 2.8% vs 18.2 ± 2.2%) | (p < 0.001) |
| Baranda (2005) | SLE | Increased levels of IL-15 was detected in serum from active and inactive patients compared with HC | See study Fig. 1A | (p<0.02) |
|  |  | Monocytes from active SLE patients  expressed higher levels of membrane-bound IL-15 compared with both inactive patients and HC | See study Fig. 3 | (p<0.05) |

**Legend:** SSc = Systemic Sclerosis, HC = Healthy Controls, VC = Vital Capacity, DLCO = diffusing capacity of lung for carbon monoxide, BD = Behcets Disease, SLE = Systemic Lupus Erythematosus, RA = Rheumatoid Arthritis, SpA = Seronegative Spondyloarthropathy, OA = Osteoarthritis, PsA = Psoriatic Arthritis, SS = Sjogrens Syndrome, FS = Focus Score, GC = Germinal Center, GPA = Granulomatosis with Polyangiitis, NK = Natural Killer, SGEC = Salivary Gland Epithelial Cells, TLR2 =Toll-Like Receptor 2

**References:**

Wuttge DM, Wildt M, Geborek P, Wollheim FA, Scheja A, Akesson A. Serum IL-15 in patients with early systemic sclerosis: a potential novel marker of lung disease. Arthritis Res Ther. 2007;9(5):R85. doi: 10.1186/ar2284. PMID: 17784951; PMCID: PMC2212554.

Gholijani N, Ataollahi MR, Samiei A, Aflaki E, Shenavandeh S, Kamali-Sarvestani E. An elevated pro-inflammatory cytokines profile in Behcet's disease: A multiplex analysis. Immunol Lett. 2017 Jun;186:46-51. doi: 10.1016/j.imlet.2016.12.001. Epub 2016 Dec 6. PMID: 27939191.

Choe JY, Lee H, Kim SG, Kim MJ, Park SH, Kim SK. The distinct expressions of interleukin-15 and interleukin-15 receptor α in Behçet's disease. Rheumatol Int. 2013 Aug;33(8):2109-15. doi: 10.1007/s00296-013-2705-4. Epub 2013 Feb 17. PMID: 23417200.

Hamzaoui K, Hamzaoui A, Ghorbel I, Khanfir M, Houman H. Levels of IL-15 in serum and cerebrospinal fluid of patients with Behçet's disease. Scand J Immunol. 2006 Dec;64(6):655-60. doi: 10.1111/j.1365-3083.2006.01844.x. PMID: 17083622.

Madej M, Nowak B, Świerkot J, Sokolik R, Chlebicki A, Korman L, Woytala P, Lubiński Ł, Wiland P. Cytokine profiles in axial spondyloarthritis. Reumatologia. 2015;53(1):9-13. doi: 10.5114/reum.2015.50551. Epub 2015 Apr 9. PMID: 27407219; PMCID: PMC4847310.

Chan A, Filer A, Parsonage G, Kollnberger S, Gundle R, Buckley CD, Bowness P. Mediation of the proinflammatory cytokine response in rheumatoid arthritis and spondylarthritis by interactions between fibroblast-like synoviocytes and natural killer cells. Arthritis Rheum. 2008 Mar;58(3):707-17. doi: 10.1002/art.23264. PMID: 18311795.

Kane D, Gogarty M, O'leary J, Silva I, Bermingham N, Bresnihan B, Fitzgerald O. Reduction of synovial sublining layer inflammation and proinflammatory cytokine expression in psoriatic arthritis treated with methotrexate. Arthritis Rheum. 2004 Oct;50(10):3286-95. doi: 10.1002/art.20518. PMID: 15476228.

Sisto M, Lorusso L, Lisi S. TLR2 signals via NF-κB to drive IL-15 production in salivary gland epithelial cells derived from patients with primary Sjögren's syndrome. Clin Exp Med. 2017 Aug;17(3):341-350. doi: 10.1007/s10238-016-0429-y. Epub 2016 Jun 3. PMID: 27260411.

Reksten TR, Jonsson MV, Szyszko EA, Brun JG, Jonsson R, Brokstad KA. Cytokine and autoantibody profiling related to histopathological features in primary Sjogren's syndrome. Rheumatology (Oxford). 2009 Sep;48(9):1102-6. doi: 10.1093/rheumatology/kep149. Epub 2009 Jul 2. PMID: 19574472.

de Menthon M, Lambert M, Guiard E, Tognarelli S, Bienvenu B, Karras A, Guillevin L, Caillat-Zucman S. Excessive interleukin-15 transpresentation endows NKG2D+CD4+ T cells with innate-like capacity to lyse vascular endothelium in granulomatosis with polyangiitis (Wegener's). Arthritis Rheum. 2011 Jul;63(7):2116-26. doi: 10.1002/art.30355. PMID: 21484763.

Pacheco-Lugo L, Sáenz-García J, Navarro Quiroz E, González Torres H, Fang L, Díaz-Olmos Y, Garavito de Egea G, Egea Bermejo E, Aroca Martínez G. Plasma cytokines as potential biomarkers of kidney damage in patients with systemic lupus erythematosus. Lupus. 2019 Jan;28(1):34-43. doi: 10.1177/0961203318812679. Epub 2018 Nov 19. PMID: 30453818.

Lin SJ, Kuo ML, Hsiao HS, Lee PT, Chen JY, Huang JL. Activating and inhibitory receptors on natural killer cells in patients with systemic lupus erythematosis-regulation with interleukin-15. PLoS One. 2017 Oct 12;12(10):e0186223. doi: 10.1371/journal.pone.0186223. PMID: 29023581; PMCID: PMC5638402.

Lin SJ, Chen JY, Kuo ML, Hsiao HS, Lee PT, Huang JL. Effect of Interleukin-15 on CD11b, CD54, and CD62L Expression on Natural Killer Cell and Natural Killer T-Like Cells in Systemic Lupus Erythematosus. Mediators Inflamm. 2016;2016:9675861. doi: 10.1155/2016/9675861. Epub 2016 Oct 26. PMID: 27847409; PMCID: PMC5101392.

Baranda L, de la Fuente H, Layseca-Espinosa E, Portales-Pérez D, Niño-Moreno P, Valencia-Pacheco G, Abud-Mendoza C, Alcocer-Varela J, González-Amaro R. IL-15 and IL-15R in leucocytes from patients with systemic lupus erythematosus. Rheumatology (Oxford). 2005 Dec;44(12):1507-13. doi: 10.1093/rheumatology/kei083. Epub 2005 Oct 26. PMID: 16251219.
