# Supplementary material for: Pathogenesis and Treatment of T-Large Granular Lymphocytic Leukemia (T-LGLL) in the Setting of Rheumatic Disease
Source: Front Oncol. 2022 Jun 7;12:854499. doi: 10.3389/fonc.2022.854499 (PMC9209697; doi:10.3389/fonc.2022.854499)
Supplement: Supplementary file 3 [file Table_2.docx]

| Study | Disease | IL-15 Relevance | Results | p=value |
| --- | --- | --- | --- | --- |
| Coit (2021) | jSSc | Enriched gene methylation in jSSc, including *STAT3*, NF-κB, and IL-15 pathways compared to HC | See study Fig. 5 | (p < 0.05) |
| Clark (2015) | SSc | Profiling of dermal blister fluid showed increased IL-15 compared to HC | 6.27 pg/ml (4.21 to 9.34) vs HC: 4.38 pg/ml (3.2 to 4.95) | (p = 0.03) |
| Wuttge (2007) | SSc | Increased serum IL-15 levels were associated with reduced nailfold capillary density | Serum IL-15 levels with reduced nailfold capillary (0.72 (0.49 to 0.90) pg/ml) compared with remaining patients (0.47 (0.27 to 0.61) pg/ml) | (p = 0.010) |
|  |  | Serum IL-15 levels correlated inversely with systolic blood pressure | 95% CI: -0.29 (-0.49 to -0.10) | (p=0.006) |
| Islam (2018) | BD | BD symptoms in mice improved after treatment with IL-15/15Rα expression vector or IL-15/IL-15Rα-Fc protein complex. | See study Fig. 4C | (p = 0.016) |
|  |  | In granulocytes, frequencies of IL-15Rα+  cells from active BD patients were down-regulated than those from HC | BD: 4.45 ± 2.89  HC: 7.78 ± 5.62%) | (p = 0.01) |
|  |  | Proportions of NK cells representing CD56+ cells were lower in active BD patients compared to HC | Active BD: 38.90 ± 17.89%  HC: 53.72 ± 15.93% | (p = 0.007) |
| Choe (2013) | BD | Serum IL-15 levels are significantly associated with ESR but not CRP | See study Fig. 2a | (r = 0.405, p = 0.027) |
|  |  | There was no difference in serum levels of IL-15 between active and inactive BD | See study Fig. 2d | (p = 0.869) |
| Curnow (2008) | BD | Levels of IL-15 in serum were increased in BD patients compared with HC | BD: 86 ± 32 pg/ml  HC: 3.4 ± 2.3 pg/ml | (p = 0.0001) |
| Hamzaoui (2006) | BD - CNS | Elevated levels of IL-15 were observed in CSF samples from neuro-BD patients and inflammatory neurological disease patients compared with patients with non-inflammatory neurological disease. | Neuro-BD patients: (11 pg/ml; 8.5-15)  Inflammatory neuro disease: (10 pg/ml; 6.5-14)  Non-inflammatory neuro disease: (4 pg/ml; 4-5.5) | (p < 0.001) |
| Han (2018) | SpA | Correlation of percentages of regulatory T cells & serum IL-15 levels between patients with AS and HC were analyzed, but no statistically significant differences were found. | See study Table 3 | (p=0.602) |
| Ciccia (2015) | AS | Increased IL-15 staining was present in the ileal biopsies of patients with AS compared to HC | See study Fig.2 | (p<0.05) |
| Tang (2013) | PsA | IL-15 is upregulated in the synovial tissue of patients with PsA but not AS, shown by immunohistochemical staining | See study Fig.4A |  |
| Bikker (2010) | SS | IL-7–stimulated cytokine production by PBMCs from patients with primary SS | IL-15 level with no stimulation: 0.70 (0.60–0.80) pg/mL  IL-15 level with IL-7 Stimulation: 0.90 (0.72–1.18) pg/mL | (p=0.003) |
| Szodoray (2005) | SS | Increased IL-15 levels in SS patients compared to HC | No statistical difference between IL-15 levels in (Germinal Cell) GC+ and GC- patients | GC+:  (p = 0.026)  GC-:  (p = 0.014) |
| Chai (2020) | IgA Vasculitis,  HSP | Frequency of NK cells was reduced in HSP patients | See study Fig. 1 | (p < 0.05) |
| Hudspeth (2019) | SLE | NK cells expressing Ki67 are increased in SLE compared to HC | See study Fig. 1 a,b | (p<0.0001) |
|  |  | High frequencies of Ki67+ NK cells are associated with increased disease severity | See study Fig. 3 | (p <0.0001) |
|  |  | Patients with dsDNA or ENA autoantibodies had higher Ki67+ NK cells compared to those without these autoantibodies | See study Fig. 4 | dsDNA: (p= 0.0247)  ENA abs: (p = 0.0026) |
|  |  | High frequencies of Ki67+ NK cells correlate with serum interleukin IL-15 levels. | See study Fig. 6 | (p=0.008) |
| Rekik (2018) | SLE | IL-15 levels were not different between SLE and HC | Data not shown | (p > 0.05) |
|  |  | IL-15 plasma concentration was decreased in *active* SLE patients | See study Fig. 4A | (p = 0.008) |
| Zhu (2016) | SLE | IL-15 mRNA was elevated in SLE compared with HC | See study Fig. 7b | p<0.05 |
| Ichinose (2015) | SLE - CNS | IL-15 was elevated in neuropsychiatric lupus compared to multiple sclerosis. | See study Fig. 1 | (p<0.0001) |
| H Bo (2009) | SLE | The level of IL-15 expression in macrophages, activated T cells & B cells from SLE male mice was increased compared with that from SLE female mice or young males. | See study Fig. 6 | (p < 0.05) |
|  |  | Recombinant hIL-15Ra-Fc IV led to decreased levels of serum dsDNA as well as proteinuria | See study Fig. 7 | (p < 0.05 in both cases) |
| Baranda (2005) | SLE | Increase in the expression of IL-15 was observed in monocytes activated with interferon in HC and inactive SLE patients compared with non-stimulated cells. | See study Fig. 1 A, B | (p<0.05) |
|  |  | The percentage of lymphocytes expressing  IL-15R-alpha was lower in inactive and active patients compared to HC | See study Fig. 3 | (p<0.05) |
|  |  | Diminished induction of CD69 by IL-15 was seen in  both inactive and active patients compared with HC | See study Fig. 4 | (p<0.05) |
|  |  | When monocytes were stimulated with IL-15, a significant increase in IFN- synthesis was observed in SLE patients and HC. | See study Fig. 5 | (p<0.05) |
| Robak (2002) | SLE | Serum levels of IL-15 were low and not significantly different in SLE patients compared to HC | SLE patients: median 2.9 pg/ml HC: median 1.6 pg/ml | (p > 0.05) |

**Legend:** jSSc = Juvenile Systemic Sclerosis, SSc = Systemic Sclerosis, HC = Healthy Controls, CI = Confidence Interval, BD = Behcets Disease, ESR = Erythrocyte Sedimentation Rate, CRP = C-reactive Protein, CNS = Central Nervous System, SpA = Seronegative Spondyloarthropathy, AS = Ankylosing Spondylitis, PsA = Psoriatic Arthritis, SS = Sjogren’s Syndrome, PBMCs = peripheral blood mononuclear cells, GC = Germinal Center, HSP = Henoch Schoenlein Purpura, NK = Natural Killer, dsDNA = double stranded deoxyribonucleic acid, ENA = Extranuclear Antigen, IFN = interferon, SLE = Systemic Lupus Erythematosus

**References:**

Coit P, Schollaert KL, Mirizio EM, Torok KS, Sawalha AH. DNA methylation patterns in juvenile systemic sclerosis and localized scleroderma. Clin Immunol. 2021 Jul;228:108756. doi: 10.1016/j.clim.2021.108756. Epub 2021 May 13. PMID: 33992755; PMCID: PMC8206011.

Clark KE, Lopez H, Abdi BA, Guerra SG, Shiwen X, Khan K, Etomi O, Martin GR, Abraham DJ, Denton CP, Stratton RJ. Multiplex cytokine analysis of dermal interstitial blister fluid defines local disease mechanisms in systemic sclerosis. Arthritis Res Ther. 2015 Mar 23;17(1):73. doi: 10.1186/s13075-015-0575-8. PMID: 25885360; PMCID: PMC4411924.

Wuttge DM, Wildt M, Geborek P, Wollheim FA, Scheja A, Akesson A. Serum IL-15 in patients with early systemic sclerosis: a potential novel marker of lung disease. Arthritis Res Ther. 2007;9(5):R85. doi: 10.1186/ar2284. PMID: 17784951; PMCID: PMC2212554.

Islam SMS, Choi B, Choi J, Lee ES, Sohn S. Frequencies of IL-15Rα+ cells in patients with Behçet's disease and the effects of overexpressing IL-15Rα+ on disease symptoms in mice. Cytokine. 2018 Oct;110:257-266. doi: 10.1016/j.cyto.2018.01.010. Epub 2018 Feb 1. PMID: 29396044.

Choe JY, Lee H, Kim SG, Kim MJ, Park SH, Kim SK. The distinct expressions of interleukin-15 and interleukin-15 receptor α in Behçet's disease. Rheumatol Int. 2013 Aug;33(8):2109-15. doi: 10.1007/s00296-013-2705-4. Epub 2013 Feb 17. PMID: 23417200.

Curnow SJ, Pryce K, Modi N, Knight B, Graham EM, Stewart JE, Fortune F, Stanford MR, Murray PI, Wallace GR. Serum cytokine profiles in Behçet's disease: is there a role for IL-15 in pathogenesis? Immunol Lett. 2008 Nov 16;121(1):7-12. doi: 10.1016/j.imlet.2008.07.009. Epub 2008 Aug 13. PMID: 18706446.

Hamzaoui K, Hamzaoui A, Ghorbel I, Khanfir M, Houman H. Levels of IL-15 in serum and cerebrospinal fluid of patients with Behçet's disease. Scand J Immunol. 2006 Dec;64(6):655-60. doi: 10.1111/j.1365-3083.2006.01844.x. PMID: 17083622.

Han R, Yang X, Chen M, Zhang X, Yuan Y, Hu X, Wang M, Liu R, Ma Y, Yang J, Xu S, Shuai Z, Jiang S, Pan F. Changes and clinical significance of CD8+CD122+ T cells in the peripheral blood of patients with ankylosing spondylitis. Clin Rheumatol. 2018 Mar;37(3):639-646. doi: 10.1007/s10067-017-3887-z. Epub 2017 Nov 6. PMID: 29110110.

Ciccia F, Guggino G, Rizzo A, Saieva L, Peralta S, Giardina A, Cannizzaro A, Sireci G, De Leo G, Alessandro R, Triolo G. Type 3 innate lymphoid cells producing IL-17 and IL-22 are expanded in the gut, in the peripheral blood, synovial fluid and bone marrow of patients with ankylosing spondylitis. Ann Rheum Dis. 2015 Sep;74(9):1739-47. doi: 10.1136/annrheumdis-2014-206323. Epub 2015 Apr 22. PMID: 25902790.

Tang F, Sally B, Ciszewski C, Abadie V, Curran SA, Groh V, Fitzgerald O, Winchester RJ, Jabri B. Interleukin 15 primes natural killer cells to kill via NKG2D and cPLA2 and this pathway is active in psoriatic arthritis. PLoS One. 2013 Sep 25;8(9):e76292. doi: 10.1371/journal.pone.0076292. PMID: 24086722; PMCID: PMC3783406.

Chan A, Filer A, Parsonage G, Kollnberger S, Gundle R, Buckley CD, Bowness P. Mediation of the proinflammatory cytokine response in rheumatoid arthritis and spondylarthritis by interactions between fibroblast-like synoviocytes and natural killer cells. Arthritis Rheum. 2008 Mar;58(3):707-17. doi: 10.1002/art.23264. PMID: 18311795.

Bikker A, van Woerkom JM, Kruize AA, Wenting-van Wijk M, de Jager W, Bijlsma JW, Lafeber FP, van Roon JA. Increased expression of interleukin-7 in labial salivary glands of patients with primary Sjögren's syndrome correlates with increased inflammation. Arthritis Rheum. 2010 Apr;62(4):969-77. doi: 10.1002/art.27318. PMID: 20131250.

Szodoray P, Alex P, Jonsson MV, Knowlton N, Dozmorov I, Nakken B, Delaleu N, Jonsson R, Centola M. Distinct profiles of Sjögren's syndrome patients with ectopic salivary gland germinal centers revealed by serum cytokines and BAFF. Clin Immunol. 2005 Nov;117(2):168-76. doi: 10.1016/j.clim.2005.06.016. Epub 2005 Aug 26. PMID: 16126006.

Chai W, Wang X, Wang W, Wang H, Mou W, Gui J. Decreased glycolysis induced dysfunction of NK cells in Henoch-Schonlein purpura patients. BMC Immunol. 2020 Oct 9;21(1):53. doi: 10.1186/s12865-020-00382-9. PMID: 33036556; PMCID: PMC7547466.

Hudspeth K, Wang S, Wang J, Rahman S, Smith MA, Casey KA; Autoimmunity Molecular Team, Manna Z, Sanjuan M, Kolbeck R, Hasni S, Ettinger R, Siegel RM. Natural killer cell expression of Ki67 is associated with elevated serum IL-15, disease activity and nephritis in systemic lupus erythematosus. Clin Exp Immunol. 2019 May;196(2):226-236. doi: 10.1111/cei.13263. Epub 2019 Mar 19. PMID: 30693467; PMCID: PMC6468178.

Rekik R, Smiti Khanfir M, Larbi T, Zamali I, Beldi-Ferchiou A, Kammoun O, Marzouki S, Hamzaoui S, Mrad S, Barbouche MR, Houman MH, Ben Ahmed M. Impaired TGF-β signaling in patients with active systemic lupus erythematosus is associated with an overexpression of IL-22. Cytokine. 2018 Aug;108:182-189. doi: 10.1016/j.cyto.2018.04.011. Epub 2018 Apr 21. PMID: 29684755.

Zhu H, Mi W, Luo H, Chen T, Liu S, Raman I, Zuo X, Li QZ. Whole-genome transcription and DNA methylation analysis of peripheral blood mononuclear cells identified aberrant gene regulation pathways in systemic lupus erythematosus. Arthritis Res Ther. 2016 Jul 13;18:162. doi: 10.1186/s13075-016-1050-x. PMID: 27412348; PMCID: PMC4942934.

Ichinose K, Arima K, Ushigusa T, Nishino A, Nakashima Y, Suzuki T, Horai Y, Nakajima H, Kawashiri SY, Iwamoto N, Tamai M, Nakamura H, Origuchi T, Motomura M, Kawakami A. Distinguishing the cerebrospinal fluid cytokine profile in neuropsychiatric systemic lupus erythematosus from other autoimmune neurological diseases. Clin Immunol. 2015 Apr;157(2):114-20. doi: 10.1016/j.clim.2015.01.010. Epub 2015 Feb 3. PMID: 25656641.

Bo H, Wei XQ, Dong H, Zhang Y, Lv P, Liu W, Koutoulaki A, Gao XM. Elevated expression of transmembrane IL-15 in immune cells correlates with the development of murine lupus: a potential target for immunotherapy against SLE. Scand J Immunol. 2009 Feb;69(2):119-29. doi: 10.1111/j.1365-3083.2008.02197.x. PMID: 19170964.

Baranda L, de la Fuente H, Layseca-Espinosa E, Portales-Pérez D, Niño-Moreno P, Valencia-Pacheco G, Abud-Mendoza C, Alcocer-Varela J, González-Amaro R. IL-15 and IL-15R in leucocytes from patients with systemic lupus erythematosus. Rheumatology (Oxford). 2005 Dec;44(12):1507-13. doi: 10.1093/rheumatology/kei083. Epub 2005 Oct 26. PMID: 16251219.

Robak E, Robak T, Wozniacka A, Zak-Prelich M, Sysa-Jedrzejowska A, Stepien H. Proinflammatory interferon-gamma--inducing monokines (interleukin-12, interleukin-18, interleukin-15)--serum profile in patients with systemic lupus erythematosus. Eur Cytokine Netw. 2002 Jul-Sep;13(3):364-8. PMID: 12231481.
